# Mixed method evaluation of a community-based physical activity program using the RE-AIM framework: Practical application in a real-world setting

**DOI:** 10.1186/s12889-015-2466-y

**Published:** 2015-11-06

**Authors:** Harriet Koorts, Fiona Gillison

**Affiliations:** Centre for Physical Activity and Nutrition Research, Deakin University, Burwood, VIC 3125 Australia; Department for Health, University of Bath, Bath, BA2 7AY UK

**Keywords:** Physical activity, Program evaluation, RE-AIM

## Abstract

**Background:**

Communities are a pivotal setting in which to promote increases in child and adolescent physical activity behaviours. Interventions implemented in these settings require effective evaluation to facilitate translation of findings to wider settings. The aims of this paper are to i) present findings from a RE-AIM evaluation of a community-based physical activity program, and ii) review the methodological challenges faced when applying RE-AIM in practice.

**Methods:**

A single mixed-methods case study was conducted based on a concurrent triangulation design. Five sources of data were collected via interviews, questionnaires, archival records, documentation and field notes. Evidence was triangulated within RE-AIM to assess individual and organisational-level program outcomes.

**Results:**

Inconsistent availability of data and a lack of robust reporting challenged assessment of all five dimensions. Reach, Implementation and setting-level Adoption were less successful, Effectiveness and Maintenance at an individual and organisational level were moderately successful. Only community-level Adoption was highly successful, reflecting the key program goal to provide community-wide participation in sport and physical activity.

**Conclusions:**

This research highlighted important methodological constraints associated with the use of RE-AIM in practice settings. Future evaluators wishing to use RE-AIM may benefit from a mixed-method triangulation approach to offset challenges with data availability and reliability.

**Electronic supplementary material:**

The online version of this article (doi:10.1186/s12889-015-2466-y) contains supplementary material, which is available to authorized users.

## Background

Current World Health Organization (2010) and UK (2011) physical activity guidelines recommend school-aged children spend a minimum of 60 min per day engaging in at least moderate intensity physical activity. At least three times a week, vigorous intensity activities should be incorporated that include muscle and bone strengthening exercises [1, 2]. In the UK during 2012 however, only 21 % of boys and 16 % of girls aged 5 to 15 years old met these daily guidelines. This is a decline from 28 % and 19 % in 2008, for boys and girls respectively [3]. Since a population shift in child and adolescent physical activity may help curb the detrimental health impact of physical inactivity in later life, there is mounting pressure for strategies which lead to a sustained increase in physical activity amongst this population.

Communities are a pivotal setting in which to promote increases in child and adolescent physical activity due to the potential high reach within this context. However, a number of factors limit our knowledge of the public health impact of community-based multicomponent physical activity interventions. Firstly, many efficacy evaluations have a weak study design [4] rendering findings relating to the long-term maintenance of such interventions inconclusive [5, 6]. Secondly, few studies directly assess factors associated with program reach, feasibility and place within an organisation or community as standard practice, omitting valuable information on program uptake and delivery [7]. Intervention studies often fail to report on generalisability characteristics [7, 8] or to specifically address the application and public health impact of the findings to real-world settings [9]. Thus, consistent evidence for the wide-scale and effective dissemination of evidence-based physical activity interventions into practice remains limited [10].

One way in which the evaluation and reporting of community-based interventions could be improved is through the use of robust evaluation models such as the RE-AIM framework (Reach, Effectiveness, Adoption, Implementation and Maintenance) [11]. RE-AIM is a widely accepted model which frames strategies to design, implement and evaluate research. RE-AIM has been successfully used to evaluate dissemination efforts of physical activity interventions in primary schools [12], using web-based technologies [13] and within the community [14, 15]. Information on the appropriate application of RE-AIM for intervention design, planning and dissemination exists [16]; yet, there are fewer discussions surrounding the benefits and challenges of applying frameworks such as RE-AIM in community-based physical activity intervention research.

NICE guidelines (2007) stipulate that effective behaviour change interventions require careful planning, design and evaluation, ideally incorporating evidence based on RCTs, high quality meta-analyses, systematic reviews of RCTs or controlled clinical trials [17, 18]. Appraising evidence to these standards is highly advocated for effective impact assessment; however, in reality, community-level interventions are not always consistently designed and implemented with such rigor. Assessing the potential public health impact of community-based interventions implemented outside of recommended guidelines is equally as important to ascertaining their potential public health impact. The lessons learned from such evaluations may improve our understanding of the wide-scale translation of both RCTs and community-based approaches and assist implementers in the design and conduct of such initiatives.

The aims of this paper are to i) present findings from a case study evaluation of a community-based physical activity intervention for children and adolescents within the UK using the RE-AIM framework (i.e., reporting outcomes of the program’s reach, effectiveness, adoption, implementation and maintenance), and ii) review the methodological challenges of conducting a robust evaluation of an existing community-based program using RE-AIM.

## Methods

### Program overview

The community-based physical activity program was available to children and adolescents aged 7–14 years residing within the local council district area. The program was promoted via on-site flyers and posters, through the program website, leaflets in community organisations (such as local supermarkets) and via word of mouth. The program set four core aims: to create ‘participation pathways’ (i.e., opportunities within the organisation that allow for continued sports participation and skill progression from early childhood into adolescence and adulthood) from ages 4–18 years in all sports included within the program, engage with the community using students as positive role models, encourage participation in sport and physical activity, and provide a fun and safe environment for young people to enjoy sport and maintain an active lifestyle. Attendees could participate in multiple sports and there was no minimum attendance period. During 2009–2010 when this evaluation took place, the program received partial financial support indirectly via National Lottery funding allocated by Sport England to the host site’s sports centre. As this funding stream was finite, a secondary aim was to generate revenue from the sports sessions to maintain its sustainability in the community. The program is unique in that it operates as part of a broader participation pathway which provides routes into performance-specific and participation-only pathways interchangeably. The participation pathway provides individuals with access to sporting facilities throughout their life with the long-term goal of contributing to a healthier lifestyle.

At the time of this evaluation, the program offered athletics, badminton, football, hockey, judo, multi-skills, netball, swimming, tennis and trampolining sessions. These activities were offered at a central hub (leisure facility), and as part of an outreach arm that delivers sporting opportunities in local schools, and provides expertise and support to local sports clubs. Hub-based sports are delivered through daily after-school sport sessions on a term-time basis and holiday sports camps during the school half-term breaks, and six week summer holiday. The aim was to mimic the school term times to maximise the program’s reach. The program had existed within the community for 7 years prior to the evaluation taking place.

The evaluation was based on a single mixed-method case study using a concurrent triangulation design [19]; triangulation is a method of comparing and contrasting multiple data sources, research methods or inferences to strengthen the validity of the interpretations [20]. The core premise is that all methods have inherent biases and limitations, therefore the use of only one method to assess a given phenomenon will inevitably yield biased and limited results [21]. As there are often multiple dimensions within a single case study, triangulation is a valuable method of corroborating evidence of the same phenomenon by viewing it from different perspectives, rather than converging on a single consistent account of the event [22]. Between-method triangulation was used within this case study to confirm the findings generated through one particular method by another. Integrating different methodologies in this way can improve the study’s validity, and overcome the biases inherent with quantitative and qualitative methodologies alone.

Due to the complexity and multiple components within the program, a mixed method approach was chosen to facilitate greater validity of inferences and more comprehensive and insightful evaluation [23]. Four embedded units of analysis were specified across two levels within the case. At the organisational level, units of analysis included the program managers (with overarching responsibility for the program) and the program coaches (delivering the sports sessions). At the individual level, units of analysis included parents (whose children attended the program) and children and adolescents who directly participated. Five sources of mixed method data were collected over 12 months which included archival records; program-related documentation; field notes from direct observations; interviews with the program managers, coaches and parents; and questionnaires from the children and adolescents attending. Qualitative and quantitative data were collected concurrently and analysed independently in short succession to avoid major developments or changes to the case.

### Participant recruitment

Ethical approval for this study was granted by the University of Bath Ethics Committee and organisational consent was obtained from the program managers.

#### Interview participants

The three program managers and all 14 program coaches (head and senior coaches) were specifically targeted for recruitment due to their seniority level within the program. This strategy ensured key informants with experience of all 10 sports were included and individuals would have sufficient knowledge of the program to contribute evidence across all five RE-AIM dimensions. Staff were invited via email to participate in a 1-hour semi-structured interview to discuss their experiences and perspectives of the program. Parents of children attending the program were recruited opportunistically via letters of invitation distributed at the main program reception desk and at the end of sports sessions. Based on initial responses, the participants of sports without parent representation were contacted directly via on-site visits, and parents asked verbally to participate. The objective was to maximise parent representation across the program sports and minimise the potential effects of volunteer bias. Signed consent was obtained prior to interview commencement.

#### Child and adolescent participants

All children and adolescents attending the program during the data collection phase were eligible to take part. Letters were sent home to parents seeking passive consent for their child’s participation 4 weeks ahead of data collection. Questionnaires were completed at the end of sports sessions over a 2-week period in February 2010. Program attendees could participate in multiple sports and therefore complete multiple questionnaires unique to each activity. Questionnaires were anonymous and participants were able to opt out on the day of data collection.

### Measurement tools

#### Interview schedule

The interview schedule contained 45 questions based on criteria within the five RE-AIM dimensions. The questions were framed in context of the program and the terminology tailored for the context of the managers, coaches or parents involved.

#### Questionnaires

A questionnaire lasting approximately 15 min was designed to assess the determinants of children’s and adolescents’ participation in the program, and factors influencing their sustained engagement. Each scale contained 21-items rated on a 5-point Likert scale ranging from 1 “unimportant” to 5 “very important”. Questions were based on environmental/organisational factors (i.e. facilities), social factors (i.e. family, peers), intrapersonal factors (i.e. goals, progression) and interpersonal factors (i.e. beliefs) as they map to the specified elements of the socioecological model [24, 25]. The reliability of the scales was established based on alpha coefficients, mean inter-item correlations and participation to item ratios.

#### Documentation

Internal program documentation describing the history and development of the program was requested monthly from the program managers and coaches. Attendance records were provided for one specific time point during data collection; February 2010. As program participation rates were transient throughout the year, the February attendance records were requested to correspond with the associated questionnaire data collection among the children and adolescents. External program documentation (i.e. online promotional material) was searched for weekly via the internet. Field notes were recorded throughout the 12-months data collection, i.e. during the interview and questionnaire data collection phases and impromptu site visits to gain a more reflective account of implementation in the natural context in which it occurred.

Additional evidence included 7 program-related documents: program advertising leaflets, posters and holiday camp brochures (*N* = 4), email correspondence with program managers outlining the program’s history and development (*N* = 1) and participant attendance records for February 2010 (*N* = 2). Archival records included local council census data containing local population figures and school statistics. Field notes were taken following 20 informal observations during the delivery of program sessions, data collection phases and from personal reflections of the program.

### Data analysis

Interviews were transcribed verbatim, transcripts entered into NVivo8 and analysed using a framework approach [26]. Framework analysis uses a hierarchical thematic framework to classify and organise data based on key themes, concepts and emergent categories [27]. Following the preliminary stage of familiarisation, transcripts were systematically indexed using codes which mapped against the RE-AIM criteria for the five dimensions. The data were then charted individually for each participant and interpreted within-and-between sub-groups until a consensus on themes was reached. To increase transferability [28, 29] of the interview data, questions were framed around the RE-AIM criteria for all five dimensions and refined following expert review of content validity.

Questionnaire data was analysed using SPSSv14 and descriptive statistics reported. Mean item scores were produced for each sport independently, stratified by participant gender and age. As the data was non-independent, figures reported relate to the number of *completed questionnaires* by age and gender, not the *number of participants* in the sample.

### Data synthesis

The interview data provided the greatest coverage of all five RE-AIM dimensions therefore initial synthesis was undertaken using this source. Firstly, the interview data was triangulated *within* each participant sub-group (program managers, coaches and parents). The strength of convergence was determined based on the frequency and extensiveness of overlapping themes. Themes were ranked across the participant group to identify those with the greatest vs the least convergence. It was expected that within-sub-groups, participants would have a similar experience and understanding of the case due to their position and level within it, therefore a similar emergence of themes was expected. Triangulation of interviews was then repeated using these emergent themes *between* participant sub-groups to identify differences *across* the case. Convergence between-groups was established when at least 2 of the 3 participant sub-groups referred to a theme. As the program managers, coaches and parents represented different levels within the case, they were not expected to have a similar experience of the program. Instances of evidence divergence were therefore expected and reported.

Evidence from the remaining four data sources was then additionally integrated into each dimension where applicable and triangulated with the existing interview themes. Questionnaire data contributed to assessing Reach, Effectiveness and Implementation. Documentation assisted with the assessment of Reach, Effectiveness, Adoption and organisational-level Maintenance. Population census data was used to assess Reach and Adoption, and field notes provided evidence for all five RE-AIM dimensions. (Additional file 1: Table S1) presents a summary of the evidence used to assess each RE-AIM dimension.

### Dimension assessment score

Following triangulation of all five data sources within each RE-AIM dimension, an individual ‘success score’ was allocated to each dimension (1 = less successful, 2 = moderately successful and 3 = highly successful). The success score was based on (i) *Data applicability* (i.e. the extent that the available data could address the dimension criteria), and (ii) *Dimension outcome* (i.e. the positive/negative outcome based on assessment of the dimension criteria using the available data).

For example Reach (*What is the absolute number, proportion and representativeness of individuals willing to participate?*): the assessment was based on whether the data available (i.e. program attendance records and interview data on uptake) could assist in quantifying the number of individuals willing to participate (success criterion i) and secondly whether the program had attained a positive outcome in terms of its Reach, defined in this case as whether the individuals included within the evaluation were representative or not of the target population based on the triangulation of participant accounts (success criterion ii). A single score was given to each dimension combining these considerations, as the interaction of data applicability and outcome meant that a high score in one would be meaningless (in terms of data quality) without a high score in the other.

## Results

Three program managers (1 male, 2 female), 3 head coaches (3 female), 4 senior coaches (1 male, 3 female) and 10 parents (1 male, 9 female) participated in an interview. The coaches represented 7 of the 10 sports (hockey, multi-skills and netball excluded) and the parents had a combined experience of 8 of the 10 sports (multi-skills and netball excluded). Parents represented 15 program attendees aged between 7 and 14 years old (7 boys, 8 girls) who had participated in the program for between 2 months to 6 years. Additional file 2: Table S2 presents the constructs highlighted during framework analysis.

Approximately 409 children and adolescents participated in the program during February 2010. In total, 334 questionnaires were completed (boys completed 181 and girls completed 153) and the mean (SD) age of participants was 9.69 (+1.88) years. Questionnaire reliability was established from Alpha coefficients which exceeded 0.70, the mean inter-item correlations exceeded 0.30 and the participant to item ratio ranged from 5:1 (for 11–14 year olds) to 11:1 (for 7–10 year olds); supporting the reliable use of the scales within this study. Results following synthesis of the data against the RE-AIM criteria is presented in Additional file 3: Table S3.

### Reach: Success score 1 (less successful)

The program reached approximately 2.5 % (*N* = 409) of the total eligible population of 6–15 year olds living within the local council district (*N* = 16,062).^1^ Interview, questionnaire, documentary data and field notes revealed attendees were more likely to be physically active or previously engaging in sports, and from a white, middleclass background than non-attenders. Evidence for the less successful reach of the program was consistent across all five data sources. The greatest depth of information gained during interviews and from field notes, the weakest source of information was obtained from internal program documentation.

### Effectiveness: Success score 2 (moderately successful)

Effectiveness was assessed based on the achievement of the program aims and objectives, reported program strengths and weaknesses, perceived outcomes following participation and the overall perceived success. Questionnaire data revealed coach rapport and ability, skill development, and improvement in health and fitness goals were important to attendee’s participation. This was concordant with interview data which identified the program as highly effective based on the social, psychological and physical benefits from participation, and absence of any negative consequences or adverse outcomes. Interviews with program managers and coaches, and internal program documentation, supported that the program objectives were perceived to have been met and criteria for program success achieved. However, the lack of formal evaluation procedures within the program limited assessment of individual-level impact. Through interviews, the managers and coaches defined program success based on the retention of attendees; yet data relating to the recruitment and retention of participants was inconsistently available.

### Adoption: Community level success score 3 (highly successful), Setting level success score 1 (less successful)

Adoption was estimated based on (i) the extent that the individual program sports adhered to the program principles and (ii) the proportion of schools and organisations within the local council district that had established links with the program. Adoption at the community-level was extremely high as links and/or partnerships were established with 95 % (*N* = 60 primary, *N* = 18 secondary) of the schools within the local education authority. Program adoption at the setting-level varied. Consistent with field notes, during interviews the coaches and parents identified weaknesses with adherence to the program principles, such as the creation of participation pathways in all program sports; which were described as inconsistently available. Data on organisations approached by the program, including those who declined uptake, was not systematically recorded. This lack of internal program documentation meant interview data was the dominant source of evidence for setting-level adoption.

### Implementation: Success score 1 (less successful)

Implementation was assessed on the basis of the consistent delivery of program components as intended, impact of program implementers and changes to the program over time. Field notes and interviews with program managers, coaches and parents revealed the aims, objectives and consistent delivery of program sports varied greatly. The program was perceived by parents and coaches to lack unity due to independent implementation of the sports, differing objectives of the sports, mixed ability, motivation and changeover of coaches. The coaches perceived these inconsistencies as potentially reducing the program’s impact on children’s progression and enjoyment of the sessions. Questionnaire data identified that the delivery, consistency and group dynamics of the sessions as important to the children’s and adolescent’s participation.

### Maintenance: Success score 2 (moderately successful)

Maintenance was assessed based on interviewee’s descriptions of retention, institutionalisation of the program within the host site and the maintenance of community links. Interview data and documentation consistently showed the program was institutionalised as part of the host’s program of community sport (i.e. documentation outlining newly formed links with local educational sites and a position within a local sports academy). Attendees typically joined at a young age and sustained participation within multiple sports for several years. However, formal rates of attrition were not available nor program documentation to assist this assessment. Attendees who were more likely to drop out were consistently described by program managers and coaches as ‘less sporty’, less competitive within sport, and from families with less support for the program. Documentation confirmed that pathways into community sport that young people could follow after the program existed, but this did vary across sports. Nonetheless, the inconsistent availability of community sports pathways after the program was identified by parents in interviews and in field notes as a barrier to children’s ongoing activity participation. Interview data and field notes highlighted parents’ lack of awareness of program pathways, despite that documentation supported their existence and availability.

## Discussion

This paper presents findings from a RE-AIM evaluation of a community-based physical activity program aimed at children and adolescents. The study had two aims, firstly to evaluate the program according the RE-AIM criteria through the triangulation of data of different formats, and secondly, to provide a critique of the challenges and facilitators of this evaluation approach in practice. Implementation of the RE-AIM framework to evaluate the program indicated that the program had only limited success in terms of Reach, Implementation and setting-level Adoption. It was highly successful in terms of community-level Adoption whereas only moderately successful in terms of Effectiveness and Maintenance; at both an individual and organisational level.

The feasible adoption of the program within the local community and its demonstrated sustainability at an organisational-level, is comparable with previous community-based interventions aimed at reducing obesity (C.H.A.M.P) [30] and increasing physical activity (JUMP-in) [31] in children. In these interventions, strong community partnerships were identified as vital to overall program success [30] and important to organisational level maintenance [31]. Whilst individual-level estimations of program Reach and Effectiveness were compromised in the current case study due to data limitations, the finding that more active children from wealthier middle class backgrounds were likely to be reached is consistent with similar intervention studies. In a systematic review of physical activity interventions in youth, across two studies in France and the United States that reported the representativeness of recruited participants compared to non-participants; non-participants were more likely to live in a low socioeconomic environment and less likely to participate in sports clubs and/or spend greater time in sedentary activities [32].

Despite challenges identifying the individual-level impact of the program, evidence for successful integration and institutionalisation of the program in the community remains an important indicator for future expansion or replication. Our findings revealed that community partnerships and adoption were integral to perceived program success, prioritised above evidence of positive participant outcomes. While this propensity emphasizes how intervention efficacy is insufficient in isolation to predict any long-term public health impact [33], it also flags up the potential risk that ineffective programmes, or those that may have a negative impact (for example, on health inequalities), may become adopted simply as they are feasible to adopt and deliver. Similarly, intervention efficacy at an individual level is not sufficient to predict successful replication or sustainability in alternate settings. Feasible program adoption and transferability to other settings remain important predictors of successful real-world replication, effectiveness and sustainability [34, 35].

While data quality and availability limited the rigour with which some elements of the framework could be assessed through a single indicator, greater confidence in these conclusions was achieved through triangulating different data sources. Assessment of all the available data ultimately led to a consistent outcome for each RE-AIM dimension, however, triangulating multiple sources provided greater clarity to instances of evidence divergence. For example, assessment of organisational-level maintenance revealed parents perceived community pathways as unavailable, yet documentation supported the expansion and availability of such community links. Rather than revealing contradictorily data, this finding highlighted an underlying lack of communication within the program regarding such pathways which emerged as a divergence of evidence.

Our second aim was to explore the methodological challenges to using the RE-AIM framework to evaluate an existing community-based physical activity initiative. To achieve the maximum informative potential of RE-AIM, assessment of all five dimensions is advocated [35, 36]. However, the lack of robust and objective data collected as part of standard monitoring practice, in addition to inconsistencies in the collection and monitoring of reporting mechanisms limited assessment of all five RE-AIM dimensions in this case.

The absence of reliable data in the program reflects a common constraint within community-based intervention evaluation. Previous evaluations of community-based interventions have reported assessment limitations to include a lack of baseline data and challenges with the organisational context [37], a lack of procedural documentation [38] and a lack of standardised implementation [39]. In general, health promotion interventions (i.e., commissioned by health services, rather than community enterprise organisations as in this case) have a greater consistency in addressing internal program delivery factors such as outcomes and attrition, yet factors influencing uptake, impact and sustainability of interventions remain infrequently reported [9, 35, 40, 41]. Data relating to individual-level factors and setting-level criteria were particularly limited within the program, such as participation rates, while information on community-level Adoption such as delivery within schools was particularly high. The lack of objective program data at the individual-level may be a result of the disparity between the public health impact-related definition of success adopted by RE-AIM, and the program managers’ and coaches’ perception of success to include community participation; prioritising engagement with external organisations. This may account for the more accurate and consistent data recorded in relation to community-level Adoption.

The lack of objectively measured outcome data may also be a consequence of conflicts between the public health-related objectives and the underpinning financial drivers of delivering a sustainable physical activity program. It has previously been suggested that appraisal of evidence should consider whether the outcome variables address the interests of the important stakeholders, and not just those who appraise the evidence [42]. According to Rychetnik et al. (2002), stakeholders include those with responsibility for implementation decisions and those affected by the intervention. Although the broad program goal was to promote community-wide participation in physical activity and sport, the sustainability of the program was ultimately dependant on income generated from participation fees. The distinction between internal program objectives and external evaluation criteria raises important questions about the applicability of RE-AIM in practice settings.

To ensure the framework criteria are addressed in sufficient depth to ascertain impact, significant consideration needs to be given to the context within which the program occurs and the underlying drivers that mediate its existence. The inclusion of setting-specific criteria within the RE-AIM framework, for example the requirement for sustainable income generation in the present case, is pivotal to its utility in practice-based and community contexts. The inclusion of only individual-level impact criteria to define effectiveness may be sufficient for estimating health impact, but it is likely that organisational-level success factors take precedence in practice. Guidance on the ways community-driven outcomes can be used as additional indicators of program effectiveness at both individual- and organisation-levels may facilitate more meaningful application of RE-AIM in practice.

Despite difficulties implementing RE-AIM due to variations in the quality and availability of setting-level data, using mixed-methods to populate the framework enhanced the richness and contextual relevance of the overall evaluation [36]. For example, the collection of multiple data sources to evaluate the same domain reduced the limitations associated with relying one data source, but also provided insight into why performance in domains was poor. A key strength of the RE-AIM framework is that it informs evaluators on which intervention elements to address for effective evaluation, rather than a prescriptive approach as to how or what process to take. Thus the setting and stakeholder priorities can be incorporated into the evaluation plan and the criteria against which the program is assessed. This can allow for a very broad and diverse methodological approach to assess the evaluation criteria, which in real-world intervention contexts is paramount to maximising the available evidence.

Collecting multiple sources of data across multiple levels strengthened the conclusions drawn from this case study when faced with the challenges of data limitations. Triangulation of the evidence enabled conflicts and consistencies to emerge and be addressed in a transparent and systematic fashion. This enabled a more in-depth understanding of factors which mediate program impact. These aspects are fundamental to the transferability of research to other settings [42]. The greatest divergence of evidence emerged between the program managers’ accounts and that with other participants and sources of data within the case. This is not surprising due the manager’s public-facing profile and awareness of the program at a predominantly senior level. Incidences of evidence divergence were as equally as informative as instances of convergence. They highlighted not only individual perceptions of the program, but their comprehension, communication and position as a program authority. The interview data was extremely rich in information and a vital source to understand why different accounts of the program existed, helping to overcome weaknesses with data unavailability from other sources. Therefore, smaller quantities of higher quality data may be of greater use and importance in real-world evaluations than larger quantities of poorly monitored and collected information.

## Strengths and limitations

### Data availability

The main limitation during assessment of all dimensions was the absence of formal monitoring data that mapped against RE-AIM recommendations. Data on the target population, eligible population, uptake and attrition were not systematically prioritised and recorded. In other cases, outcomes did not map to RE-AIM guidelines due to practical issues. For example, Implementation could not be assessed based on costing information as recommended within RE-AIM, due to the complex financial and organisational structure of the program; total program delivery cost was unavailable as some of the sports were managed and partly funded as independent entities.

### Program standardisation

The program was delivered in the community as a single organisation, yet the sports implemented within it had varying implementation procedures and their own additional program objectives. The lack of internal program standardisation challenged assessment of all five RE-AIM dimensions, in particular at the organisational level. Assessment of setting level Adoption was challenged by the differing autonomy of head coaches over others, making adherence to the program principles and standardisation of reporting procedures more diverse.

### Evaluation methodology

The scoring method used in this research was developed based on recommendations for using mixed methodology when applying RE-AIM in practice [36], and in context of the vast disparity of available data in this real-world situation. Assessment was therefore based on whether the dimension was achieved *and* the extent that the data available could contribute to assessing that dimension. Objectivity of ‘success’ was achieved by continually mapping the data against the RE-AIM specifications, and whilst this meant the program was evaluated in the context within which it occurred; the additional non-standardised assessment criteria may only apply to this current situation. Nonetheless, assessing RE-AIM using only the dimension criteria as the benchmark without consideration of data availability could have led to distorted conclusions of potential program impact.

The lack of availability of data experienced in this research is a true reflection of many community-based programs implemented in similar settings. The use of robust evaluation frameworks such as RE-AIM in these contexts is warranted, and recommended, however acknowledgement and inclusion of context-specific information such as data availability may be necessary to achieve more reflective interpretations.

Case study research has previously been criticised for the potential limitations associated with the generalisability of results [43], however, case studies have a unique advantage of facilitating the exploration of social phenomena in the context it naturally occurs [44]. Key informants of the program were specifically included in this study to achieve a more informed depiction of the case. To minimise the risk of social desirability and increase the research validity, participants were recruited across multiple points within the program and their evidence triangulated across participant subgroups. Nonetheless, the fact the data was weighted heavily towards interviews does invite the potential for bias, emphasising individual’s perceptions of a situation as opposed to objective records which can quantify specific events. Mixed methodology was therefore a major strength within this research as the weaknesses of one methodology can be offset by the strengths of another [45]. Collecting both qualitative and quantitative data in this evaluation increased the validity and reliability of the results, as a more rigorous assessment of differing perspectives was achieved.

## Conclusion

This study adds knowledge to existing methodologies used to implement RE-AIM in the community and facilitates discussion of possible solutions to the methodological challenges experienced in practice settings. Funding organisations are increasingly acknowledging the importance of health as an outcome in addition to sports participation (i.e. Sport England, 2014), and thus the ability to provide robust evidence that meets the needs of different audiences is increasing [42, 46]. This research has highlighted important methodological challenges associated with the use of RE-AIM in community-based practice settings. It also provides insight into the role of triangulation of mixed methods to assist real-world intervention evaluations when preferred outcome data is absent or inconsistent. Future evaluators wishing to implement RE-AIM may benefit from a mixed-method triangulation approach to offset the challenges with data availability and reliability. Explicit reporting of the data limitations and gaps in dimension assessment is recommended to improve the transparency of program evaluation reporting, and aid more informed interpretation of potential program impact in practice.

## Additional files

Additional file 1:
**Overview of evidence used to assess each RE-AIM dimension.** Table demonstrating the application of different evidence sources to address each RE-AIM criteria. (DOCX 13 kb) Additional file 2: Table S2. Constructs highlighted during framework analysis. Table of identified interview themes, mapped against the RE-AIM framework dimensions. (DOCX 19 kb) Additional file 3: Table S3.
**Application of mixed method data to assess the RE-AIM dimensions.** Table presenting synthesis of data from all five data sources, mapped against the RE-AIM framework dimensions. (DOCX 15 kb)

## Abbreviation

RE-AIM: Reach, Effectiveness, Adoption, Implementation, Maintenance

## Footnotes

1 Council census data recorded children’s ages based on school year groups only. This resulted in an age grouping of 6–15 years whereas the program was eligible to only those aged 7–14 years. The proportion of the eligible population reached by the program was therefore based on estimates and reach could not be indisputably quantified.
