# Supplementary material for: Mixed method evaluation of a community-based physical activity program using the RE-AIM framework: Practical application in a real-world setting
Source: BMC Public Health. 2015 Nov 6;15:1102. doi: 10.1186/s12889-015-2466-y (PMC4635975; doi:10.1186/s12889-015-2466-y)
Supplement: Additional file 1: — Overview of evidence used to assess each RE-AIM dimension. Table demonstrating the application of different evidence sources to address each RE-AIM criteria. (DOCX 13 kb) [file 12889_2015_2466_MOESM1_ESM.docx]

**Additional file 1**

Table 1 Overview of evidence used to assess each RE-AIM dimension

| **Data source** | **RE-AIM dimension** | | | | |
| --- | --- | --- | --- | --- | --- |
|  | **Reach** | **Effectiveness** | **Adoption** | **Implementation** | **Maintenance** |
| Interviews |  |  |  |  |  |
| Questionnaires |  |  |  |  |  |
| Documentation |  |  |  |  |  |
| Census data |  |  |  |  |  |
| Field notes |  |  |  |  |  |
