# Supplementary material for: Mixed method evaluation of a community-based physical activity program using the RE-AIM framework: Practical application in a real-world setting
Source: BMC Public Health. 2015 Nov 6;15:1102. doi: 10.1186/s12889-015-2466-y (PMC4635975; doi:10.1186/s12889-015-2466-y)
Supplement: Additional file 2: Table S2. — Constructs highlighted during framework analysis. Table of identified interview themes, mapped against the RE-AIM framework dimensions. (DOCX 19 kb) [file 12889_2015_2466_MOESM2_ESM.docx]

**Additional file 2**

Table 2 Constructs highlighted during framework analysis

| **Framework:**  **RE-AIM** | **Program Managers** | **Program Coaches** | **Parents of program attendees** |
| --- | --- | --- | --- |
| **Reach** | (i) *Public awareness of program*   - Within schools - Links through schools and other organisations - Through the website - Word of Mouth   (ii) *Population reached*   - Physically active or sporty - Wealthy or attend a private school   (ii) *Program advertisement*   - Need to increase promotion and awareness   (iii) *Determinants of children’s participation*   - Their own decision - Influential parents - Amount of sport they have access to at school - They develop and improve in the sport and progress   (iv) *Determinants of parent’s participation*   - Compatible with lifestyle - Provides childcare - Beneficial for the child (sociability and fitness levels) | (i) *Public awareness of program*   - Word of mouth - Leaflets - Within Schools   (ii) *Population reached*   - Physically active or sporty - Wealthy or attend a private school   (ii) *Program advertisement*   - - Advertising is poor or non-existent   - No awareness of the program   - Coaches personally recruit children   - Need more promotion and awareness   (iii) *Determinants of children’s participation*   - Their own decision - Influential parents - They have friends that attend - They develop, improve in sport and progress - Become competitive and focussed - Enjoyment of the sessions   (iv) *Determinants of parent’s participation*   - Provides childcare - Beneficial for the child - Coaches and facilities | (i) *Public awareness of program*   - Word of mouth - Website   (ii) *Population reached*   - Physically active or sporty - Wealthy or attend a private school   (iii) *Program advertisement*   - - Advertising is poor or non-existent   - No active program promotion   - Not aware of advertising   (iv) *Determinants of children’s participation*   - Achievement of personal gains and progression - Become competitive and focussed in the sport - Enjoyment of the sessions - Friends that attend and the group dynamics - Amount of sport they do at school   (v) *Determinants of parent’s participation*   - Image of Team Bath as prestigious and elite - Beneficial for the child’s health, ability and sociability - Compatible to lifestyle; convenient & provides childcare - Offers value for money, affordable |
| **Effectiveness** | (i) The aims and objectives of the program   - To improve in sport and develop skills - Making sport accessible to all children - Children have fun and enjoyment - Progress through the program into elite sport   (ii) Definition of program success   - Number of program attendees - Retention of program attendees - Monetary profit gained   (iii) Strengths and weakness of the program   - Offers a range of sports - Offers non-competitive sport - Good coaching offered - Lack of alternatives in the local council district - Positive brand image   (iv) Feedback and evaluation procedures within the program   - Lack of interest among staff in session activity and delivery - Parent feedback dismissed or not taken seriously - Drop outs not recorded - Parents approach managers to provide feedback - React when sufficient number of feedback on a topic   (v) Outcomes following participation   - Increase in child’s confidence - Increased sociability with others - Improved skill and physical fitness level | (i) The aims and objectives of the program   - To improve in sport and develop skills - Enabling a range of sports to be accessible - Making sport accessible to all children - Children have fun and enjoyment - Progress through the program to elite sport   (ii) Definition of program success   - Number of program attendees   (iii) Strengths and weakness of the program   - Provides excellent facilities - Offers a range of sports - Good coaching and small groups - Lack of alternatives in the local council district - Positive brand image   (iv) Feedback and evaluation procedures within the program   - Lack of interest among staff in session activity and delivery - Parent feedback not taken seriously - Lack of or poor formal parental feedback and evaluation   (v) Outcomes following participation   - Increase in child’s confidence - Increased sociability with others - Improved skill and physical fitness level | (i) Perceptions of the program aims and objectives   - To support children to improve in sport and develop skills - Enabling a range of sports to be accessible to all children   (ii) Definition of program success   - Child’s improvement within sport   (iii) Strengths and weakness of the program   - Excellent facilities and offers and range of sports - Good coaching - Positive brand image - Lack of alternatives in the local council district   (iv) Feedback and evaluation procedures within the program   - Parental feedback and evaluation doesn’t exist or is poor - Lack of opportunity to provide feedback - Program appear disinterested in proactively seeking feedback - No follow-up of pursuit of drop outs   (v) Outcomes following child’s participation   - Increase in child’s confidence - Improved skill and physical fitness level - Increased sociability with others - Greater awareness of health and fitness - Awareness of team ethics and sportsmanship |
| **Adoption** | (i) Adoption rate of the program in the community (community-level)   - Every school in the local council district was described as linked to the program   (ii) Representativeness of program settings   - Schools with limited sports facilities and lack of funding less likely to adopt - Challenges included mixed motivations of children, large group size, difficulties with session discipline - Barriers: children don’t get a real experience of the sport | (i) Adoption rate of the program across the different sports (setting-level)   - Adoption of program principles varied - Sports adopted the program differently - Delivered sessions independently of overarching program, self-run - Program lacked unity   (ii) Adoption rate of the program in the community (community-level)   - Every school in local council district was described as linked to the program - Challenges include limited availability of clubs beyond the program   (iii) Representativeness of program settings   - Challenges included mixed motivations of children, large group size, difficulties with session discipline - Barriers: children don’t get a real experience of the sport | (i) Adoption rate of the program across the different sports (setting-level)   - Adoption of program principles varied - Sports adopted the program differently, implemented independently - Program lacked unity, no concept of the programs as a ‘whole’ - Lacked awareness of other sports available |
| **Implementation** | (i) Delivery of the sports sessions   - Lack of consistency in coaching - Unreliability of coaches/students - Conflict of student coaches with academic pressures - Aims and objectives differ across coaches - Coaches lack of or mixed experience and motivation to teach   (ii) Communication within the program   - Communication within program lacking - Improved communication and planning identified as a need   (iii) Roles and responsibilities in the program   - Rely on coaches as unavoidable lack awareness of individual sessions | (i) Delivery of the sports sessions   - Lack of consistency in coaching - Unreliability of coaches - Lack of session preparation - Aims and objectives differ across coaches - Coaches lack of or mixed experience and motivation to teach - Varied structure and aim to sessions by coaches   (ii) Communication within the program   - Poor communication with program managers, lack of interest - Weak links between program head office and lack of direction - Improved communication and planning identified as a need - Improved team work required   (iii) Roles and responsibilities in the program   - Lack of clarity who’s responsible for guidance on sessions - Promotion of sports and consistent implementation perceived as managers role - Coaches expect training from management | (i) Delivery of the sports sessions   - Lack of consistency in coaches - Unreliability of coaches - Coaches have mixed experience and ability - Inconsistent session delivery - Lack of session aims, structure, planning which varied across sports - Child groups mixed interests, abilities and ages - Whole program lacked structure and organisation   (ii) Communication within the program   - Communication with program managers poor and essential for implementation - Perceived lack of importance by managers - Lack of clarity of roles and responsibilities in the program   (iii) ‘Concept’ of the program to promote physical activity   - No ‘concept’ of the program overall - Unclear on program aims based on inconsistent implementation - Confusing brand image - Unaware of other program sports and how integrated into program |
| **Maintenance** | (i) Rates of attendance and attrition   - Participation from a young age and retention for several years - Attendance began in sports available pre-program and was sustained - Pathways between tots sessions and the program were essential - Drop outs not consistently recorded - Drop outs not perceived as large and therefore important - Children more likely to drop out: less active in sport, less competitive, from less privileged backgrounds, or from unsupportive families - Reasons for drop out external to program (i.e. age)   (ii) Institutionalisation of the program within the host site and the community   - Successfully institutionalised due to pathways between tots sessions and this program - Program established a part of host site’s program of community sport - Inconsistent links between program and community sports clubs/groups | (i) Rates of attendance and attrition   - Participation from a young age and retention for several years - Attendance began in sports available pre-program and was sustained - Pathways between tots sessions and the program were essential - Drop outs not consistently recorded - Drop outs not perceived as large and therefore important - Children more likely to drop out: less active in sport, less competitive, from less privileged backgrounds, or from unsupportive families - Some procedures in place to overcome drop outs (i.e. fees to re-join) - Reasons for drop out external to program   (ii) Institutionalisation of the program within the host site and the community   - Successfully institutionalised due to pathways between tots sessions and this program - Program established a part of host site’s program of community sport - Inconsistent links between program and community sports clubs/groups | (i) Rates of attendance and attrition   - Children participated from a young age and remained for several years - Progression from tots program key - Children who had dropped out not followed-up - Reasons for drop out included structure of sports sessions, time commitments, pressure for competition and negative group dynamics - Reasons for drop out also external to program (i.e. school workload)   (ii) Institutionalisation of the program within the host site and the community   - Successfully institutionalised due to pathways between tots sessions and this program - Lacked awareness of participation pathways after program and into community - Community pathways insufficiently promoted - Perceived lack of pathways associated with children’s ceased participation in sport |
