# Supplementary material for: Mixed method evaluation of a community-based physical activity program using the RE-AIM framework: Practical application in a real-world setting
Source: BMC Public Health. 2015 Nov 6;15:1102. doi: 10.1186/s12889-015-2466-y (PMC4635975; doi:10.1186/s12889-015-2466-y)
Supplement: Additional file 3: Table S3. — Application of mixed method data to assess the RE-AIM dimensions. Table presenting synthesis of data from all five data sources, mapped against the RE-AIM framework dimensions. (DOCX 15 kb) [file 12889_2015_2466_MOESM3_ESM.docx]

**Additional file 3**

Table 3 Application of mixed method data to assess the RE-AIM dimensions

|  | **Assessment question** | **Data source** | **Data applicability & Dimension outcome** | **Overall success score** |
| --- | --- | --- | --- | --- |
| **R** | What percent of potentially eligible participants a) were excluded, b) took part and c) how representative were they? | 1. Council census data (2009)  2. Interview Data  3. Questionnaire Data  4. Documentation *(Electronic/hard copies program advertisement, weekly attendance record)*  5. Field notes | 1. Census data related to ages 6-15yrs not 7-14yrs; population eligibility unable to be assessed.  2. Population eligible described as all children aged 7-14yrs, consensus that population reached and representativeness of attendees limited.  3. Population reached and representativeness; children’s determinants of participation included fun, health and fitness related goals.  4. Program advertisements equal representation of all sports, evidence to support all 7-14 year olds eligible. Greater advertisement at one site; limiting reach.  5. Contributed to population eligible, population reached; cost of participation associated with uptake, population eligible unclear. | **1** |
| **E** | What impact did the intervention have on a) all participants who began the program; b) on process intermediate and primary outcomes; and c) on both positive and negative (unintended), outcomes including quality of life? | 1. Interview Data  2. Questionnaire Data  3. Documentation *(Overview of history and development, weekly attendance report 2009)*  4. Field notes | 1. Assessment of program aims and objective, and outcomes; consensus that program lead to positive outcomes, no negative consequences, consistent program strengths. Definition of program success varied, no evaluation procedures to assess impact.  2. Perceived importance factors in the program; rapport and coach ability, improvement within sport, fitness and health. Consistent with interview data.  3. Aims and objectives of program consistent across documents, success criteria based on participation rates; annual attendance record exceeded targets.  4. Consistent with manager’s definition of success regarding participation rates. Program demonstrated flexibility and could adapt to demand, but lacked formal evaluation and feedback. | **2** |
| **A** | What percent of settings and intervention agents within these settings (e.g., schools/educators, medical offices/physicians) a) were excluded, b) participated and c) how representative were they? | 1. Primary and secondary school census data (2009)  2. Documentation *(‘Community links with the program’, ‘Opportunities for school years 1-6 and 7-13’, a record of delivery to Primary and Secondary schools, 2009, Overview of history and development)*  3. Interview Data  4. Field notes | 1. Links established with 95% of primary and secondary schools in the local government area; i.e. delivered sports coaching or hosted holiday camps/sports festivals with schools.  2. Supported interview data regarding aims to partner with community organisations. Since 2003, interactions and partnerships/links in the community had increased, links with community strong and well-established.  3. Community-level data from managers and coaches: consistently described as highly successful, supporting census data. Limited availability of community clubs and cost challenged adoption.  3. Setting-level data from parents and coaches: adherence to program principles varied, sports run independently; consistent adoption poor.  4. Setting level adoption varied; adherence to program principles, awareness of the program differed across sports. Consistent with interviews, sports functioned independently of the program. | **3**  (comm. level)  **1**  (setting level) |
| **I** | To what extent were the various intervention components delivered as intended (in the protocol), especially when conducted by different (non-research) staff members in applied settings? | 1. Interview Data  2. Questionnaire Data  3. Field notes | 1. Convergence of evidence: communication and organisation of coaches poor, ability and motivations of coaches differed, roles and responsibilities unclear. Parents had no concept of whole program; overall lacked consistency in implementation.  2. Perceived importance of factors associated with implementation; positive rapport with coach, feelings of inclusion in the session, timings of the session. Contributes to assessment of consistency in implementation and potential program impact.  3. Contributed to delivery of the program and organisation of coaches; disorganisation among coaches during holiday sports camps for some sports, observation that children’s experience varied greatly across the program according to the sport, parent’s lacked awareness of different program elements, cancellations inconsistently communicated. | **1** |
| **M** | a) What were the long-term effects (minimum of 6-12 months following intervention)? b) What was the attrition rate; were drop-outs representative; and how did attrition impact conclusions about effectiveness?  a) To what extent were different intervention components continued or institutionalized?  b) How was the original program modified? | 1. Interview Data  2. Documentation *(Record of delivery to Primary and Secondary schools 2009, Overview of history and development)*  3. Field notes | 1. Individual level: consensus attendees joined at a young age and participated for several years; participation pathways before program important to potential long-term program impact. Divergence evidence between managers and coaches regarding records of attrition; inconsistent evidence drop-outs monitored, parent consensus drop outs not followed-up.  1. Organisational level: consensus pathways embedded in organisation, established as part of Universities program of community sport. Inconsistent availability pathways after program and into the community. Parents unaware of community clubs; some association with child’s ceased participation in sport.  2. Documentation described participation prior to and after the program. Evidence for expansion of the program via new sports clubs and partnerships with some sports clubs in the community.  3. Parents aware of lack of non-competitive sports opportunities after program, opportunities for recreational sport after the program were perceived as limited. | **2**  (Indiv level  **2**  (Org level) |

Success score ratings; 1=Less successful, 2=Moderately successful, 3=Highly successful
